# Applications of Ti/TiO_2_ nanotube arrays/CeO_2_@PbO_2_ and Ti/β-PbO_2_-CuO_*x*_ electrodes for efficient electrocatalytic degradation of paraquat: cell design and comprehensive mechanistic studies

**DOI:** 10.1039/d5ra06796k

**Published:** 2025-10-17

**Authors:** Davood Nematollahi, Mahsa Roshani, Mohammad Mehdi Hashemi-Mashouf, Niloofar Mohamadighader

**Affiliations:** a Faculty of Chemistry and Petroleum Sciences, Bu-Ali Sina University Hamedan 65178-38683 Iran nemat@basu.ac.ir; b Plant Chemistry Research Center, Bu-Ali Sina University Hamedan Iran

## Abstract

Ti/TiO_2_ nanotube arrays (NTA)/CeO_2_@PbO_2_ and Ti/β-PbO_2_-CuO_*x*_ electrodes were used for the electrocatalytic degradation of paraquat (PQ^2+^), a highly toxic and widely used herbicide. The fabricated electrodes were characterized by XPS, SEM, EDS, mapping, XRD and EIS methods. The results show that the performance of the Ti/TiO_2_ nanotube in the electrocatalytic degradation of PQ^2+^ is better than that of the Ti/β-PbO_2_-CuO_*x*_ electrode. This electrode increases the production of hydroxyl radicals and degradation efficiency. The results show that the highest degradation efficiency of 75% was achieved at pH = 7, a current density of 7.8 mA cm^−2^ and an initial concentration of 50 ppm. The intermediate species formed during the electrolysis of PQ^2+^ were analyzed based on cyclic voltammetry, UV-vis spectroscopy and LC-MS techniques, and a possible mechanism for the degradation of PQ^2+^ was proposed. In the second part of this study, the electrochemical behavior of PQ^2+^ was studied, gaining a deeper insight and understanding of the redox properties and adsorption activity of this molecule.

## Abstract 2

Ti/TiO_2_ nanotube arrays (NTA)/CeO_2_@PbO_2_ and Ti/β-PbO_2_-CuO_*x*_ electrodes were used for the electrocatalytic degradation of paraquat (PQ^2+^), a highly toxic and widely used herbicide.

## Graphical abstract

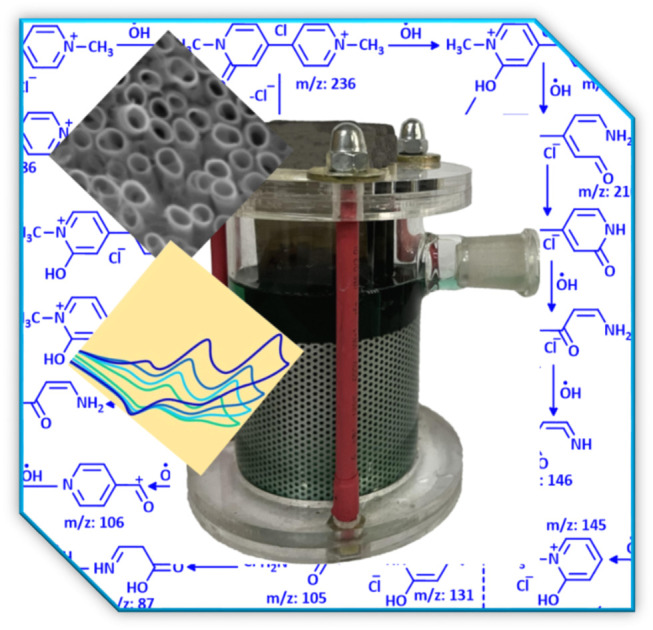

## Introduction

On average, 2 million tons of pesticides are used annually in the world to fight against pests, insects and weeds and herbicides are the most widely used type, accounting for 47.5% of global pesticide consumption. Paraquat (PQ^2+^) (1,1′-dimethyl-4,4′-bipyridinium dichloride) (also known as methyl viologen) is a non-selective broad-spectrum herbicide that has been widely used for decades. When this pollutant enters the body, it circulates through the bloodstream and is fixed in various tissues, causing serious diseases and even death. The toxicity of PQ^2+^ has been found to be related to the production of superoxide ions.^1^

Various methods have been reported for the removal of PQ^2+^ from aquatic environments. Adsorption methods are effective for the treatment of PQ^2+^. In these methods, adsorbents such as activated carbon,^2^ activated bleaching earth,^3^ modified zeolite,^4^ methacrylic acid-modified rice husk,^5^ clays^6^ and montmorillonite^7^ are typically used. The main drawback of these methods is secondary pollution. Another category of PQ^2+^ treatment methods is advanced oxidation processes (AOPs). These methods are based on the generation and use of hydroxyl radicals (a powerful oxidant) created by chemical reactions. These methods include heterogeneous photocatalytic processes in the presence of TiO_2_ (ref. 8) or UV ozonation^9^ methods for the degradation/mineralization of PQ^2+^. These methods do not have the drawbacks of the previous method due to the mineralization of organic pollutants. In addition to the above-mentioned methods, electrochemical advanced oxidation methods (EAOP), as a more progressive method, have also been used for the degradation of PQ^2+^. In these methods, the degradation of organic pollutants is done by the *in situ* electrochemical generation of hydroxyl radicals.^10^ These methods include anodic oxidation (AO),^11,12^ Fenton,^13^ electro-Fenton (EF),^11,13^ photoelectron-Fenton (PEF),^11,13^ photoelectrooxidation.^13^ Each of these methods has its own advantages and disadvantages. In addition, recently, Khlifi and colleagues used a novel palladium-based perovskite catalyst as a cathodic modifier in a boron-doped diamond (BDD)-assisted electro-Fenton system for the degradation of PQ^2+^. This electrode significantly accelerated the degradation process and led to almost complete mineralization under optimal conditions.^14^

Direct electrochemical methods using boron-doped diamond (BDD) electrodes have also been used to decompose PQ^2+^. In 2024, Teutli-Sequeira *et al.* used a commercial BDD film at circumneutral pH for PQ^2+^ mineralization due to its lower energy and fewer chemical requirements, as well as the mechanical stability of BDD electrodes.^15^ They succeeded in mineralizing PQ^2+^ by more than 90%. Also, Bautista-Garcia *et al.* (2025) performed the degradation of PQ^2+^ using the BDD–Fe system by the UVA-LED photoelectrooxidation method.^16^ Using this method, they were able to remove this herbicide with an efficiency of 77% under optimal conditions. In a comprehensive and interesting study, Rabaaoui *et al.* (2025) investigated the electrochemical degradation of PQ^2+^ using a BDD anode.^17^ They investigated the effect of operational variables, including the effect of anode material, and reported that BDD electrodes showed the most efficient performance, achieving COD and TOC removal rates of 99% and 98.6%, respectively. The results of these methods are valuable, and BDD allows for the complete mineralization of organic pollutants; however, these electrodes are very expensive, which limits their use.

In order to develop the use of efficient and cost-effective Ti/TiO_2_/CeO_2_@PbO_2_ nanotube arrays and Ti/β-PbO_2_-CuO_*x*_ electrodes for the degradation of persistent pollutants, in the present study, the electrocatalytic activity and degradation efficiency of these electrodes for the degradation of PQ^2+^ were investigated. The results show that the Ti/TiO_2_/CeO_2_@PbO_2_ nanotube has better efficiency in the degradation of PQ^2+^ than that of the Ti/β-PbO_2_-CuO_*x*_ electrode. We also designed a new electrochemical cell for the degradation of PQ^2+^. Using this cell, the degradation efficiency was increased by 95% and energy consumption was reduced by 40%. In addition, in this research, we made considerable efforts to develop more active electrode materials for the degradation of PQ^2+^ and advance the frontiers of knowledge in the field of its electrochemical properties. We believe that the outcome of this research can serve as a good guide for removing this herbicide from aquatic environments. On the other hand, the results of the electrochemical studies conducted in this research lead to a deeper insight and understanding of the redox properties and adsorption activity of PQ^2+^.

## Experimental section

### Apparatus and reagents

Chemicals used in this study, including oxalic acid, Pb(NO_3_)_2_, Ce(NO_3_)_3_, NaOH, NaF, ethylene glycol, Cu(NO_3_)_2_, HNO_3_, NH_4_F, Na_2_SO_4_, were purchased from Sigma-Aldrich and Merck. All of the chemicals were of analytical grade and used directly without further purification. Paraquat was obtained from Alborz Behsam Company, Tehran, Iran. The buffers used in this research were prepared by dissolving HClO_4_ (for pH: 1), H_3_PO_4_ (for pHs: 2, 3, 6, 7, 8), acetic acid (for pHs: 4, 5), NaHCO_3_ (for pHs: 9, 10) and Na_2_CO_3_ (for pHs: 11, 12) in deionized water and adjusting the pH value using NaOH or HCl solutions.

A model 691 pH meter (Metrohm, Switzerland) was used for pH measurements. The crystalline structure of the electrodes was investigated through X-ray diffraction (XRD, Italstructure, ADP200, Italy and GNR explorer, Italy) with a radiation source of Cu Kα in the 2*θ* range of 20 to 90 at the wavelength of 0.154 nm. The elemental chemical state was analyzed using X-ray photoelectron spectroscopy (XPS, Thermo Fisher Scientific K-ALPHA) with an Al Kα source. To evaluate the morphology of bare and modified electrodes, scanning electron microscopy (SEM, TESCAN-MIRA3-XMU) images in different magnifications were employed. For determining chemical elements and their distribution, energy-dispersive X-ray spectroscopy (EDS) and elemental mapping assay were utilized, respectively. Electrochemical degradation and electrodeposition were performed using a DC power supply (MEGATEC MP-3005, CHINA). The UV-vis absorption spectra of the solutions were obtained using an Analytik Jena Specord 210 UV-vis spectrophotometer. Organic intermediates formed during the degradation of PQ^2+^ were analyzed using liquid chromatography-mass spectrometry (LC-MS). LC-MS analysis was conducted with a Shimadzu LCMS-2010A system equipped with a Eurospher C18 column. The mobile phase consisted of a mixture of acetonitrile (ACN) with 0.1% formic acid and water with 0.1% formic acid. For this purpose, a few milliliters of the PQ^2+^ solution were collected during electrolysis and analyzed using LC-MS in a certified laboratory. Electrochemical measurements were performed using an Auto Lab®/PGSTAT30 potentiostat/galvanostat (Eco Chemie, Utrecht, The Netherlands). Electrochemical impedance spectroscopy (EIS) was performed using Zahner Zennium and Thales software (Version of 5.0.6.1). To calculate energy consumption, the same DC power supply (MEGATEC MP-3005, China) was used to apply a constant current (±3 mA) and record the cell potential (±3 mV).

The concentration of metal ions in the catalyst was evaluated using inductively coupled plasma-optical emission spectrometry (ICP-OES; 730-ES Varian).

### Electrochemical cell

**Fig. 1:**
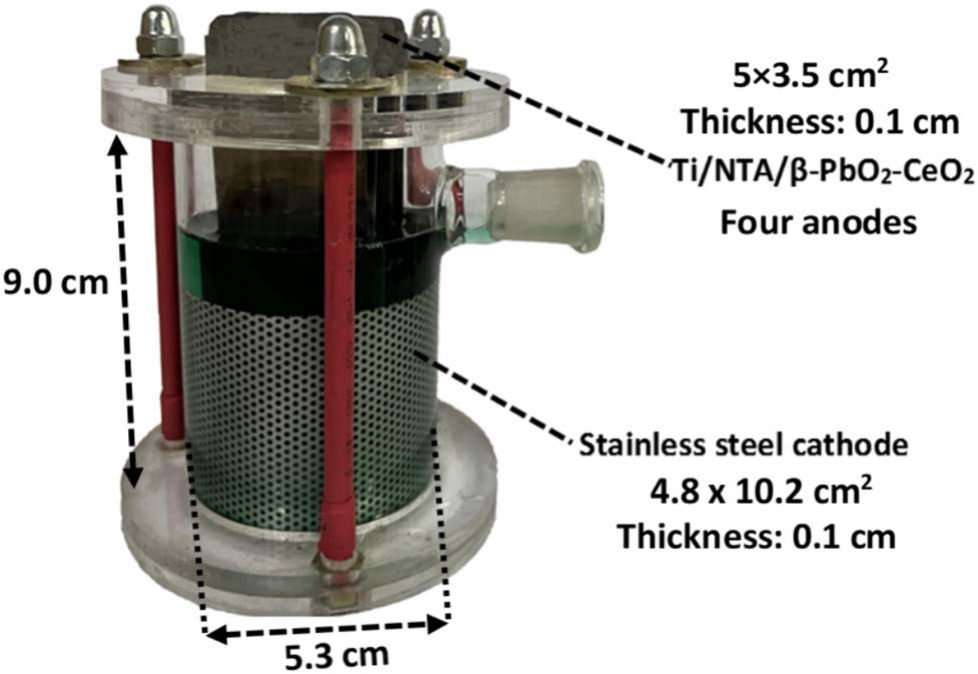
Cell design for the electrochemical degradation of PQ^2+^ using the Ti/NTA/β-PbO_2_-CeO_2_ electrode.

In this study, a new glass electrochemical cell with a volume of 200 ml was designed and built to achieve the lowest cost and highest efficiency of electrochemical degradation (Fig. 1). The prominent feature of this cell is the increase in the ratio of the electrode surface area to the cell volume, which increases the production of hydroxyl radicals, and as a result, increases the efficiency of the cell. In this cell, the cathode is a stainless steel mesh, with a thickness of 0.1 cm, a height of 5.0 cm and an area of 101 cm^2^, which is attached to the inner wall of the cell, as shown. This cell is also equipped with four plate anodes (5 × 3.5 cm^2^ and a thickness of 0.1 cm), with an area of 140 cm^2^, which are located in the center. In this cell, the distance between the electrodes is 1 cm, and a magnetic stirrer was used to facilitate convective mass transfer during the electrocatalytic degradation of PQ^2+^. This cell operates at current densities of 4.7 and 7.8 mA cm^−2^.

### Pretreatment of the titanium surface

A titanium sheet (grade 1) with dimensions of 5 cm × 2 cm × 0.1 cm with an effective surface of 21 cm was used. First, the Ti sheet was polished using sandpaper (grit 400) to obtain a smoother surface. After that, the Ti sheet was sonicated in 1 M sodium hydroxide at room temperature for 40 minutes to remove the oxides and impurities and then washed with distilled water. In the next step, the Ti sheet was immersed in boiling 30% oxalic acid for 1 hour and then washed with distilled water. The reason for using boiling oxalic acid was to effectively remove all impurities and create a sufficiently rough surface for electrochemical deposition. After that, the Ti sheet is immersed in boiling distilled water for 30 minutes to clean the surface of residual oxalic acid.^18^ Using this method, three Ti sheets were prepared as the anode substrate, which had a total effective area of 63 cm^2^.

### Preparation of Ti/β-PbO_2_-CuO_*x*_

In order to prepare Ti/β-PbO_2_-CuO_*x*_, a cell consisting of treated Ti sheets (see previous section) as anode and two stainless steel sheets as cathode, containing Pb(NO_3_)_2_ (0.5 M), Cu(NO_3_)_2_ (0.2 M), HNO_3_ (0.1 M), NaF (0.01 M) and 2.5 vol% ethylene glycol, was electrolyzed at a current density of 10 mA cm^−2^ at 65 °C for 120 min.^19^ More details on the electrode preparation method are reported in ref. 19.

### Preparation of Ti/NTA/β-PbO_2_-CeO_2_

**Fig. 2:**
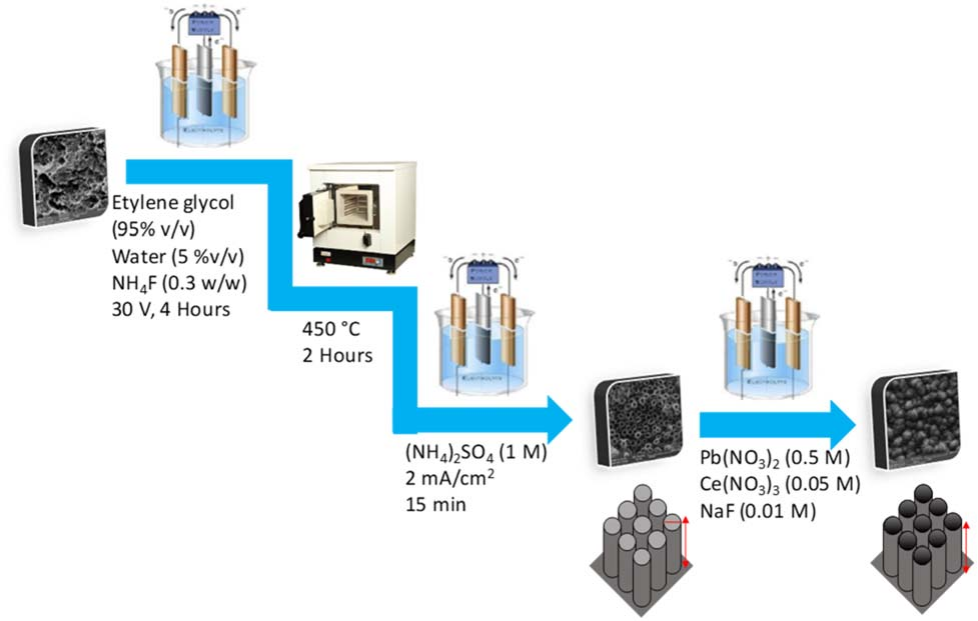
Preparation steps of the Ti/NTA/β-PbO_2_-CeO_2_ electrode.

In the first step to prepare Ti/NTA, the treated Ti sheets (see Pretreatment of the titanium surface section) as anode and stainless steel sheets (with the same dimensions) as cathode were placed alternately with a distance of 0.5 cm in a cell containing a solution of ethylene glycol-water (95% v/v) and 0.3% NH_4_F. Then, the cell was electrolyzed for 240 minutes with an applied potential of 30 V at room temperature. After that, the titanium sheets were placed in a furnace at 450 °C for 120 minutes. In the next step, the titanium sheets were electrolyzed in an electrochemical cell containing 1 M (NH_4_)_2_SO_4_ by applying a current density of 3 mA cm^−2^ for 15 minutes at room temperature.^20,21^ In the next step, Ti/NTA electrodes as anodes and stainless steel sheets as cathodes were placed in a solution containing Pb(NO_3_)_2_ (0.5 M), Ce(NO_3_)_3_ (0.004 M), NaF (0.01 M) and HNO_3_ (0.1 M) and electrolyzed at a current density of 10 mA cm^−2^ for 2 hours at room temperature.^22^ The preparation steps of the Ti/NTA/β-PbO_2_-CeO_2_ electrode are shown in Fig. 2. More details on the electrode preparation are reported in ref. 20–22.

## Results and discussion

### Electrode characterization

Understanding the structure of the electrode and tracking its structural changes during the pollutant degradation process is very important to analyze the pollutant degradation mechanism. XPS, SEM, EDS-mapping, XRD analysis and EIS were performed to investigate the electrode surface characteristics.

#### XPS analysis

**Fig. 3:**
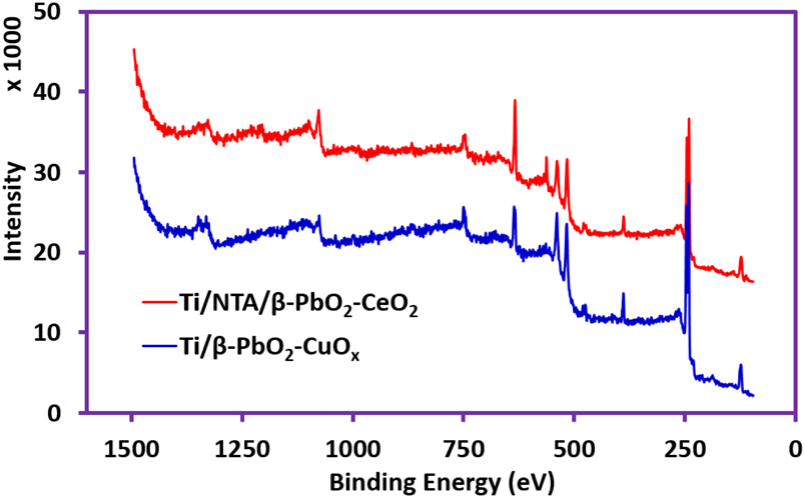
XPS survey scan (between 1500 and 0 eV) of Ti–NTA–PbO_2_-CeO_2_ and Ti/β-PbO_2_-CuO_*x*_ electrodes.

**Fig. 4:**
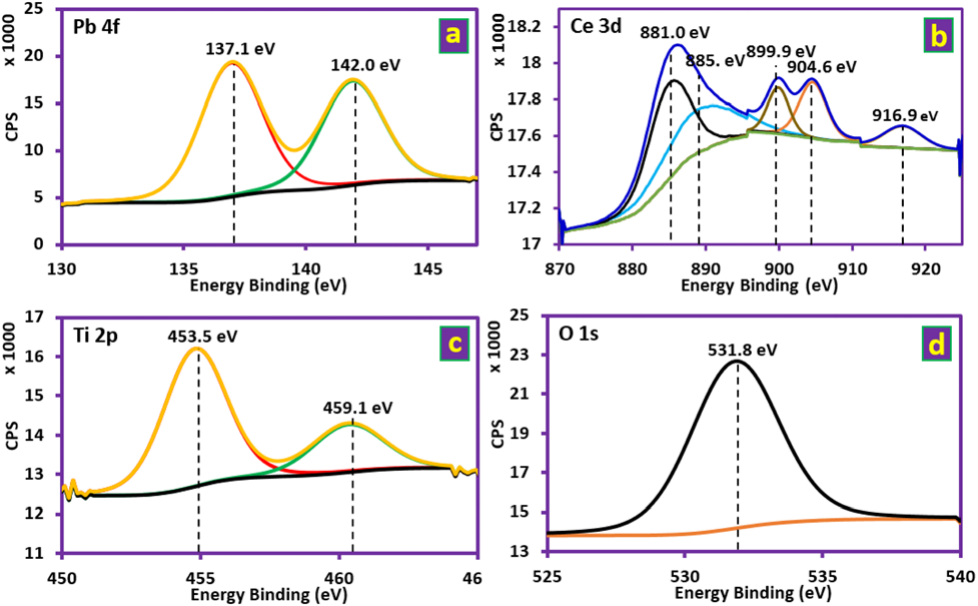
Deconvoluted XPS spectra of the Ti/NTA/β-PbO_2_-CeO_2_ electrode at Pb 4f, Ce 3d Ti 2p and O 1s core levels.

**Fig. 5:**
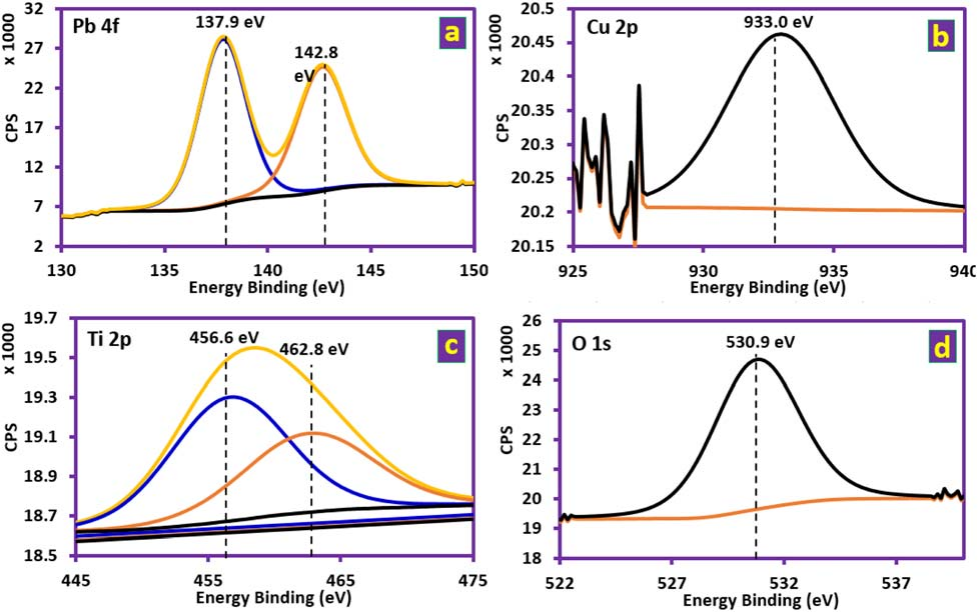
Deconvoluted XPS spectrum of the Ti/β-PbO_2_-CuO_*x*_ electrode at Pb 4f, Cu 2p, Ti 2p and O 1s core levels.

XPS analysis was performed to clarify the chemical state of Ti/NTA/β-PbO_2_-CeO_2_ and Ti/β-PbO_2_-CuO_*x*_ electrode compositions. Fig. 3 shows the survey scan of both Ti/NTA/β-PbO_2_-CeO_2_ and Ti/β-PbO_2_-CuO_*x*_ electrodes. Fig. 4 shows the high-resolution spectrum of the Ti/NTA/β-PbO_2_-CeO_2_ electrode, demonstrating the successful stabilization of the tetravalent oxidation state of Pb in the nanostructured complex. Fig. 4a displays the Pb 4f core level spectrum, with binding energies (BE) of 137.1 and 142.0 eV, corresponding to Pb 4f 7/2 and Pb 4f 5/2, respectively, with a 4.9 eV spin–orbit splitting.^23^ The Ce 3d core level spectrum (Fig. 4b) exhibits five peaks corresponding to three pairs of spin–orbit doublets. Due to the low amount of Ce in the electrode composition, one of the peaks remains unobserved.^24–26^Fig. 4c shows the Ti 2p core level spectrum, and the BE values of 453.5 and 459.1 eV correspond to Ti 2p 3/2 and Ti 2p ½, respectively, and spin–orbit splitting at 5.6 eV, indicating the oxidation state of titanium as Ti^4+^.^27^Fig. 4d shows the O 1s core spectrum with a BE of 531.8 eV, suggesting O–Pb and O–Ti bonding.^28,29^Fig. 5 shows the high-resolution spectrum of the Ti/β-PbO_2_-CuO_*x*_ electrode. The Pb 4f core level spectrum is displayed in Fig. 5a; the BE values of 137.1 and 142.0 eV correspond to Pb 4f 7/2 and Pb 4f 5/2, respectively, with a 4.9 eV splitting, which confirms the predominant phase is the β-phase.^28^Fig. 5b depicts the Cu core level spectrum with a BE of 933.0 eV. Due to the low amount of Cu in the electrode composition, spin–orbit splitting is not observed. Fig. 5c shows the Ti 2p core level spectrum, with the BE of 456.6 and 462.8 eV and a 6.2 eV splitting, showing the Ti^0^ state.^29^Fig. 5d depicts O 1s with a BE of 530.9 eV, indicative of the O–Cu bond.^30^

#### XRD analysis

**Fig. 6:**
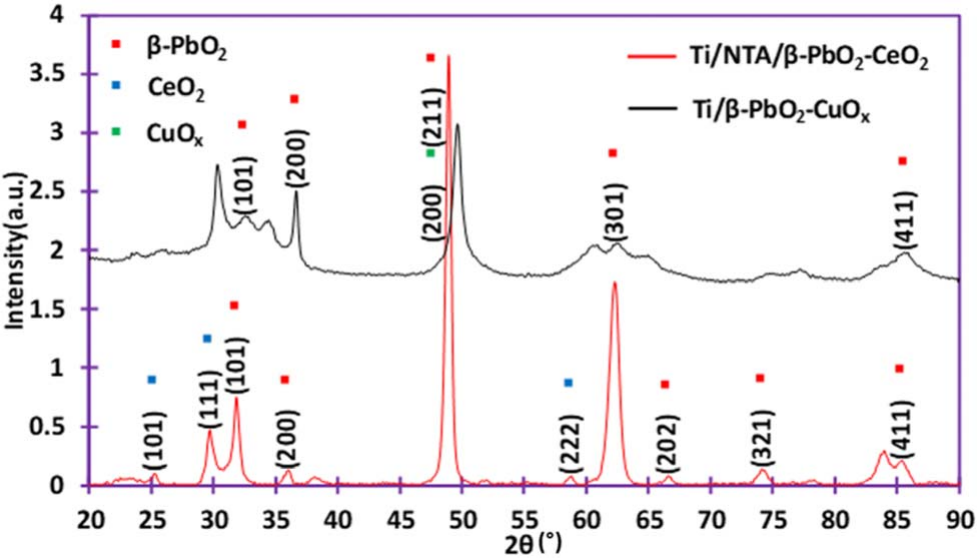
Comparison of diffractograms of Ti/NTA/β-PbO_2_-CeO_2_ and Ti/β-PbO_2_-CuO_*x*_ electrodes.

The XRD pattern of the Ti/NTA/β-PbO_2_-CeO_2_ electrode is shown in Fig. 6. The peaks with 2*θ* values of 31.85° (101), 36.06° (200), 48.93° (211), 62.30° (301), 66.58° (202), 74.12° (321) and 85.40° (411) correspond to β-PbO_2_. The peaks with 2*θ* = 29.7° (111) and 58.8° (222) correspond to CeO_2_ and the peak with a 2*θ* of 25.26° (101) corresponds to the TiO_2_ anatase phase. All peaks are in good agreement with ICSD card 89-2805.^31,32^ Ce doping with PbO_2_ may be the reason for reducing the size of PbO_2_ crystals, thereby increasing the specific surface area and electrocatalytic activity of the electrode, and can also accelerate the lifetime of the electrode.^33^ The XRD pattern of the Ti/β-PbO_2_-CuO_*x*_ electrode is also shown in Fig. 6.

The peaks with 2*θ* values of 2*θ* = 32.56° (101), 36.66° (200), 49.64° (211), 60.55° (112), 62.21° (301), 77.21° (400) and 85.88° (411) correspond to β-PbO_2_. The peak with a 2*θ* value of 2*θ* = 49.64° is attributed to the (200) plane corresponding to doped-Cu. All peaks are in good agreement with ICSD card 89-2805.^34^ Ethylene glycol (EG) has no effect on the crystal structure of β-PbO_2_; in fact, EG surrounds the PbO_2_ particles and prevents the growth of PbO_2_ crystals and in addition, EG increases nucleation.^19^ Copper reduces the crystal size. Also, copper ions prevent the deposition of metallic lead on the cathode during electrochemical deposition.^35,36^

The average crystallite sizes were estimated by the Scherrer formula:^37^

1 *D* = *Kλ*/*β* cos(*θ*)

*D* is the crystalline size (nm), *K* is Scherrer's constant (0.91), *λ* is the wavelength of radiation (0.1541874 m), *β* is the corrected half width of the diffraction peak and *θ* is the diffraction angle. The calculated average grain sizes of Ti/NTA/β-PbO_2_-CeO_2_ and Ti/β-PbO_2_-CuO_*x*_ electrodes were 14 nm and 16 nm, respectively. The results show that the Ti/NTA/β-PbO_2_-CeO_2_ electrode has a smaller crystalline size and thus a higher specific surface area, indicating better performance in electrochemical degradation.

#### SEM analysis

**Fig. 7:**
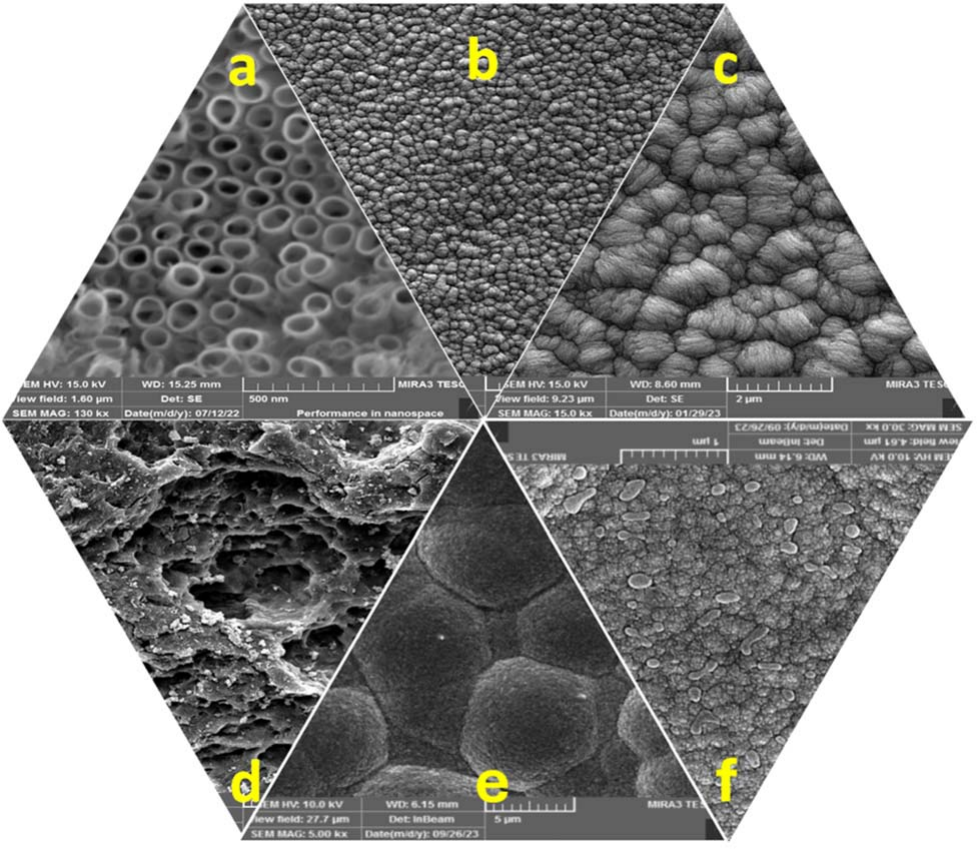
SEM images of (a) TiO_2_ nanotube arrays. (b and c) Ti/NTA/β-PbO_2_-CeO_2_ in 5 μm and 2 μm magnifications, respectively. (d) Bare Ti. (e and f) Ti/β-PbO_2_-CuO_*x*_ in 5 μm and 1 μm magnifications, respectively.

SEM images of Ti/NTAs/β-PbO_2_-CeO_2_ and Ti/β-PbO_2_-CuO_*x*_ electrodes are shown in Fig. 7. Fig. 7a shows the SEM image of the nanotubes and confirms that the Ti nanotubes completely cover the electrode surface. Ti/NTAs are promising substrates for deposition due to their large specific surface area, good conductivity, high chemical stability, and cost-effectiveness.^38^ The SEM images of Ti/NTAs/β-PbO_2_-CeO_2_ in Fig. 7b and c at different magnifications show the crystal structure of β-PbO_2_ with a uniform, crack-free, and compact cluster distribution in both electrodes.^39^ From the SEM images and XRD analysis, it is evident that Ti/NTAs/β-PbO_2_-CeO_2_ has a smaller crystalline size, which indicates a greater ability for catalytic activity and better performance in electrochemical degradation.^33^ The average inner diameter of nanotubes (using ImageJ software) was determined to be 166 nm. Fig. 7d shows the Ti (bare) electrode. Fig. 7e and f show PbO_2_ modified with copper and ethylene glycol (EG) at different magnifications. Adding copper to the composition reduces the size of the crystal structure of β-PbO_2_. By adding EG to the composition, the morphology undergoes substantial changes. The crystals transform into bulk structures, providing more space for the deposition of the modified layer, while the size of the lead dioxide particles decreases and nucleation increases. The addition of ethylene glycol with 2.5% v/v is the most optimal mode to modify the substrate surface.^19^

#### EDS and mapping analysis

**Fig. 8:**
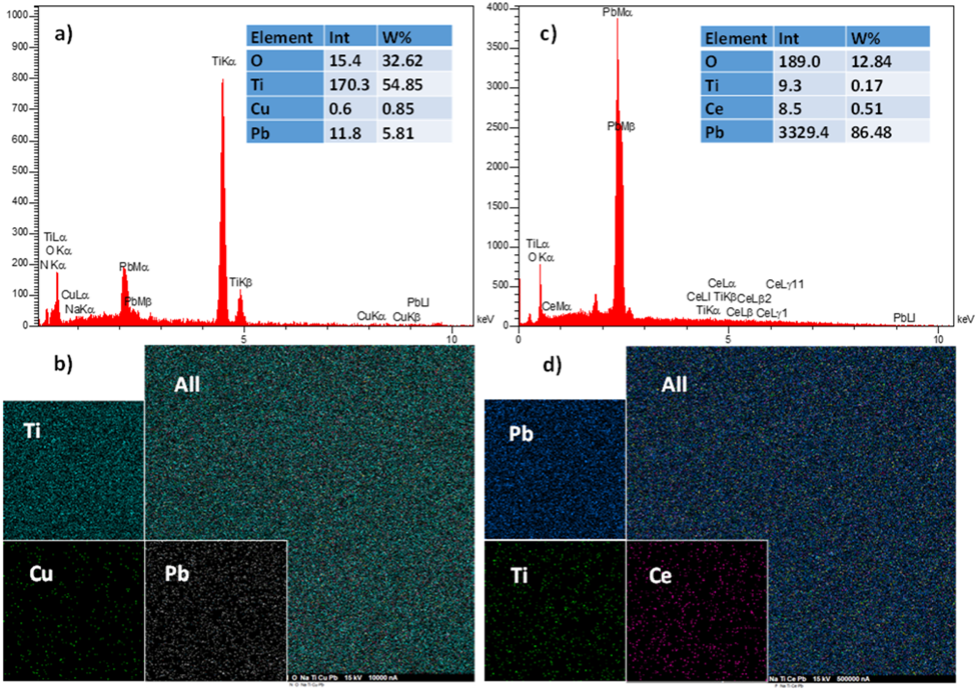
(a) EDS-elemental analysis of Ti/β-PbO_2_-CuO_*x*_. (c) EDS-elemental analysis of Ti/NTA/β-PbO_2_-CeO_2_. (b) Mapping analysis of Ti/β-PbO_2_-CuO_*x*_. (d) Mapping analysis of Ti/NTA/β-PbO_2_-CeO_2_.

The EDS pattern of Ti/β-PbO_2_-CuO_*x*_ and Ti/NTAs/β-PbO_2_-CeO_2_ electrodes are shown in Fig. 8a and c. Obviously, all elements were successfully deposited with very small amounts of Na and F elements. The addition of NaF prevents the penetration of the generated oxygen into the crystal lattice of PbO_2_.^12^ In order to determine the inner surface of nanotubes and increase the accuracy of EDS analysis, the deposition was accompanied by gold doping. The EDS pattern of the Ti/β-PbO_2_-CuO_*x*_ electrode shown in Fig. 8a confirms the presence of Pb, Cu, O and Ti elements on the electrode substrate. The absence of the carbon element indicates that EG is not deposited on the electrode surface. Mapping analysis of both electrodes (Fig. 8 parts b and d) shows complete coverage of the electrode surface by the constituent particles (as expected). It also effectively shows the dispersion and uniformity of doped elements without the accumulation of any elements in Ti/NTAs/β-PbO_2_-CeO_2_ and Ti/β-PbO_2_-CuO_*x*_ electrodes.

#### Electrochemical impedance spectroscopy

**Fig. 9:**
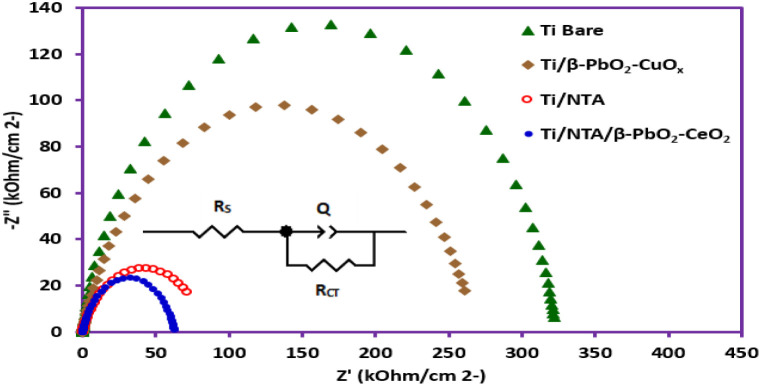
Nyquist plots of different electrodes in 0.2 M KNO_3_. Impedance spectra were measured under the open-circuit condition with an AC potential of 50 mV from 100 kHz to 0.01 Hz at room temperature. Potential applied: for Ti/β-PbO_2_-CuO_*x*_: 1.294 V. for Ti/NTA/β-PbO_2_-CeO_2_: 1.115 V. for Ti/NTA: 0.478 V and for bare Ti: 0.446 V. Electrochemical cell: undivided cell. Counter electrode: platinum wire. Reference electrode: Ag/AgCl electrode.

Electrochemical impedance spectroscopy was used to study the performance of electrodes in solution. The Nyquist plots of electrodes are shown in Fig. 9. The diameter of the capacitive loops in the Nyquist diagrams shows the charge-transfer resistance. In general, a smaller semicircle with a smaller diameter indicates a lower charge-transfer resistance.^40^ As can be seen, the charge transfer resistance in the Ti/NTA electrode is much lower than that of Ti bare and Ti/β-PbO_2_-CuO_*x*_ electrodes. A possible explanation for this behavior can be due to the increase in the active surface in the Ti/NTA and Ti/NTA/β-PbO_2_-CeO_2_ electrodes caused by the nanotubes. The equivalent circuit of the electrochemical performance of the prepared electrodes is shown in Fig. 9. In this circuit, *R*_s_ represents the solution resistance, *Q* represents the double layer capacitance and *R*_CT_ represents the charge transfer resistance. The charge transfer resistance for Ti Bare (Ti sheet), Ti/β-PbO_2_-CuO_*x*_, Ti/NTA and Ti/NTA/β-PbO_2_-CeO_2_ are 3.23 × 10^2^ kΩ, 2.68 × 10^2^ kΩ, 84.6 kΩ and 62.7 kΩ, respectively.

### Electrochemical studies of PQ^2+^

**Fig. 10:**
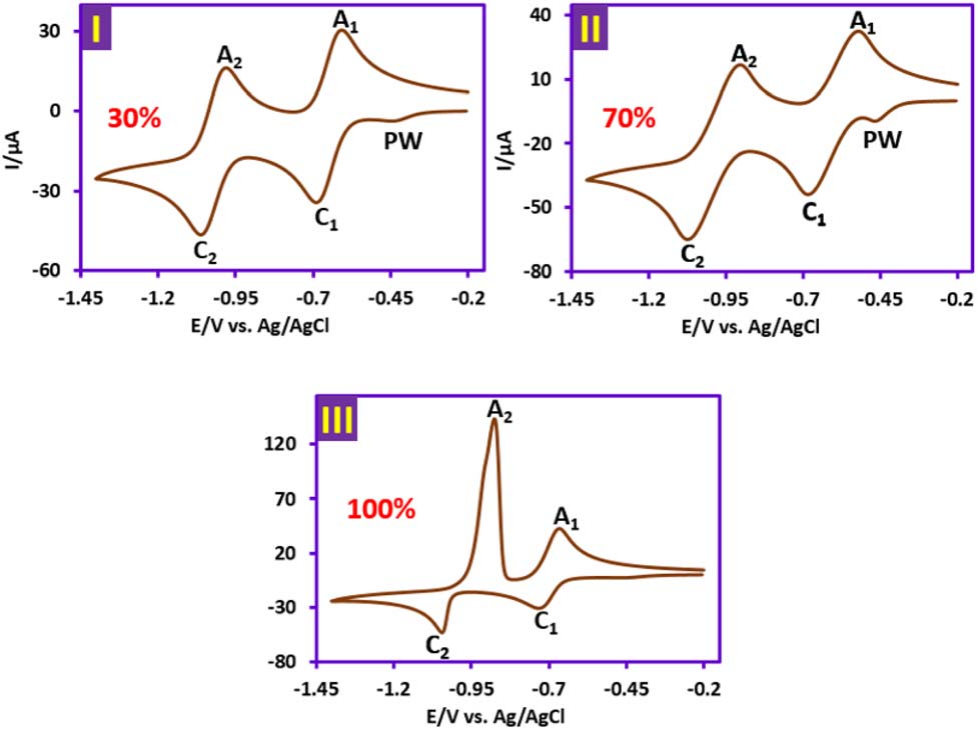
Mechanism of cyclic voltammograms of 1 mM solution of PQ^2+^ in: (I) phosphate buffer solution (pH = 7.0, *c* = 0.2 M)/acetonitrile mixture (70/30 v/v). (II) Phosphate buffer solution (pH = 7.0, *c* = 0.2 M)/acetonitrile mixture (30/70 v/v). (III) Phosphate buffer solution (pH = 7.0, *c* = 0.2 M): at a scan rate of 100 mV s^−1^ and room temperature. Electrochemical cell: undivided cell. Working electrode: glassy carbon electrode. Counter electrode: platinum wire. Potential scan range: −0.20 to −1.40 V *vs.* Ag/AgCl.

**Scheme 1:**
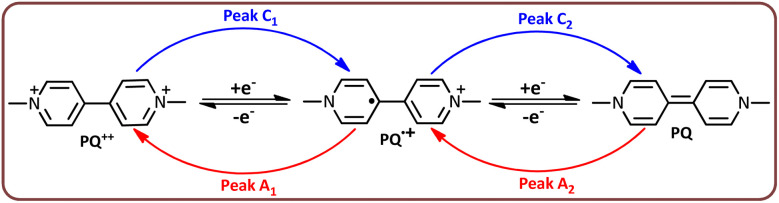
Stepwise reduction of PQ^2+^ to PQ^+^˙ and PQ.

Cyclic voltammogram of 1 mM solution of *N*,*N*′-dimethyl-4,4′-bipyridiniumdichloride (PQ^2+^) in water (phosphate buffer, pH = 7.0, *c* = 0.2 M)/acetonitrile (30/70 v/v) is shown in Fig. 10, part I. As seen in the cathodic scan, two well-defined peaks, C_1_ and C_2_, can be observed at potentials of −0.68 and −1.06 V, which are attributed to the stepwise reduction of PQ^2+^ to PQ^+^˙ and PQ (Scheme 1). In the reverse scan, two anodic peaks A_1_ and A_2_ are clearly observed at potentials of −0.61 and −0.97 V *vs.* Ag/AgCl, which are the counterparts of the cathodic peaks C_1_ and C_2_, respectively.

**Fig. 11:**
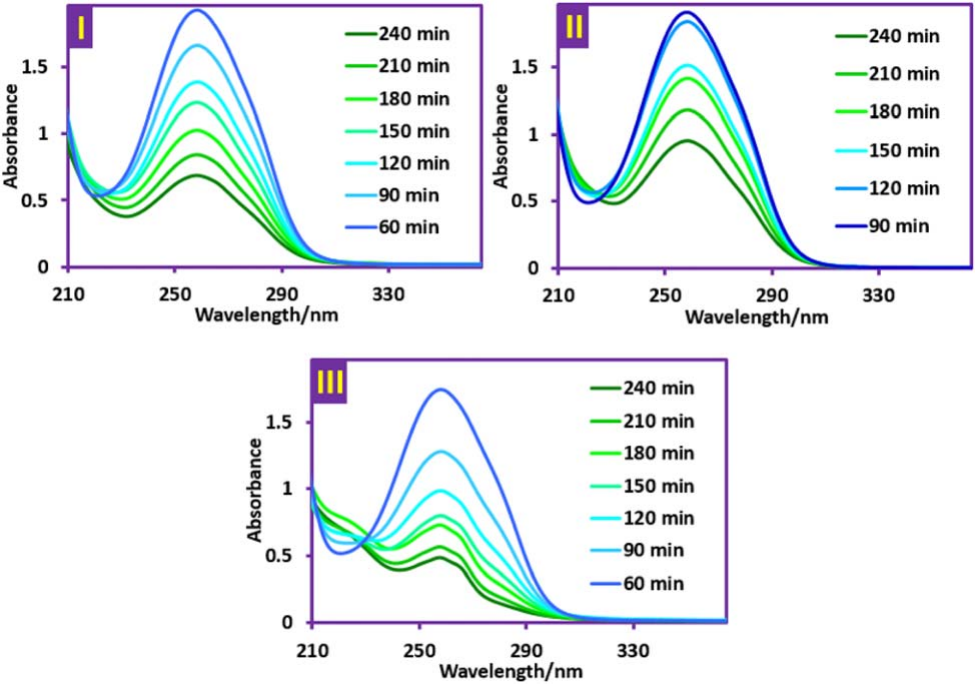
UV-vis spectra of PQ^2+^ during the degradation process. (I) Ti/NTA/β-PbO_2_-CeO_2_ electrode in a conventional cell. (II) Ti/β-PbO_2_-CuO_*x*_ electrode in a conventional cell. (III) Ti/NTA/β-PbO_2_-CeO_2_ electrode in a newly designed cell. Electrolysis condition: initial concentration of PQ^2+^ is 50 ppm, current density of 4.7 mA cm^−2^. Solvent: aqueous phosphate buffer solution (pH = 7.0, *c* = 0.2 M).

As the water ratio increases to 70%, there is no significant change in the voltammogram shape, except for a slight increase in the pre-peak (PW) current at a potential of −0.46 V (Fig. 11, part II). But in the solution without acetonitrile (100% water), the shape of the voltammogram changes significantly (Fig. 11, part III). In this condition, peak A_2_ shows a strong adsorption behavior and confirms that fully reduced paraquat (PQ) (uncharged species) is strongly absorbed in the aqueous solvent.^41^ The effect of solution pH on the adsorption process of reduced paraquat (PQ) was also studied, and it was found that such behavior is not observed in acidic environments. Protonation of nitrogen atoms in the structure of PQ in acidic solutions may be the main reason for the lack of strong adsorption of this molecule. Finally, our data show that the reduced form of this herbicide definitely has strong adsorption properties in neutral and alkaline solutions that should be considered in environmental assessments.

### Galvanostatic electrolysis of PQ^2+^

These experiments were performed with initial concentrations of 50 and 70 ppm of PQ^2+^ at current densities of 4.7 and 7.8 mA cm^−2^ and pHs of 2, 7 and 9. Monitoring of PQ^2+^ degradation was carried out using UV-vis spectrophotometry at a wavelength of 257 nm. The first experiments were performed to compare the efficiency of Ti/NTA/β-PbO_2_-CeO_2_ and Ti/β-PbO_2_-CuO_*x*_ electrodes in PQ^2+^ degradation in a conventional cell. The volume of this cell was 80 ml and equipped with 3 anodes with dimensions of 2.5 × 4.4 cm and an effective surface of 64 cm^2^ and 2 stainless steel cathodes with dimensions of 2.5 × 4.5 cm. The stirring of the solution was performed by a magnetic stirrer at a constant speed. Fig. 11, parts I and II, shows the UV-vis spectra of PQ^2+^ during its degradation using Ti/NTA/β-PbO_2_-CeO_2_ and Ti/β-PbO_2_-CuO_*x*_ electrodes, respectively. The comparison of the decrease in absorbance at the wavelength of 257 nm in Fig. 11(I) and (II) shows that the efficiency of the Ti/NTA/β-PbO_2_-CeO_2_ electrode in the degradation of PQ^2+^ is higher than that of the Ti/β-PbO_2_-CuO_*x*_ electrode; therefore, this electrode was used in the subsequent experiments in the newly designed cell. The volume of the new cell is 170 ml and equipped with four Ti/NTA/β-PbO_2_-CeO_2_ anodes with dimensions of 3.5 × 5.0 cm and an effective area of 140 cm^2^ with a distance of 1 cm from each other and a cathode made of a stainless steel mesh that the anodes are surrounded by it (Fig. 2).

Fig. 11, part III, shows the UV-vis spectrum of the degradation of PQ^2+^ using Ti/NTA/β-PbO_2_-CeO_2_ in the newly designed cell. As can be seen, the decrease in absorbance at a wavelength of 257 nm is greater than in the previous cases.

Since there is a possibility of copper, lead, and titanium ions in the solution at the end of electrolysis, the presence of these ions in the electrolyzed solution was determined using ICP-OES. The results showed that the concentration of all four ions in the solution is lower than the limit of detection of the ICP-OES instrument.

### Influence of current density

**Fig. 12:**
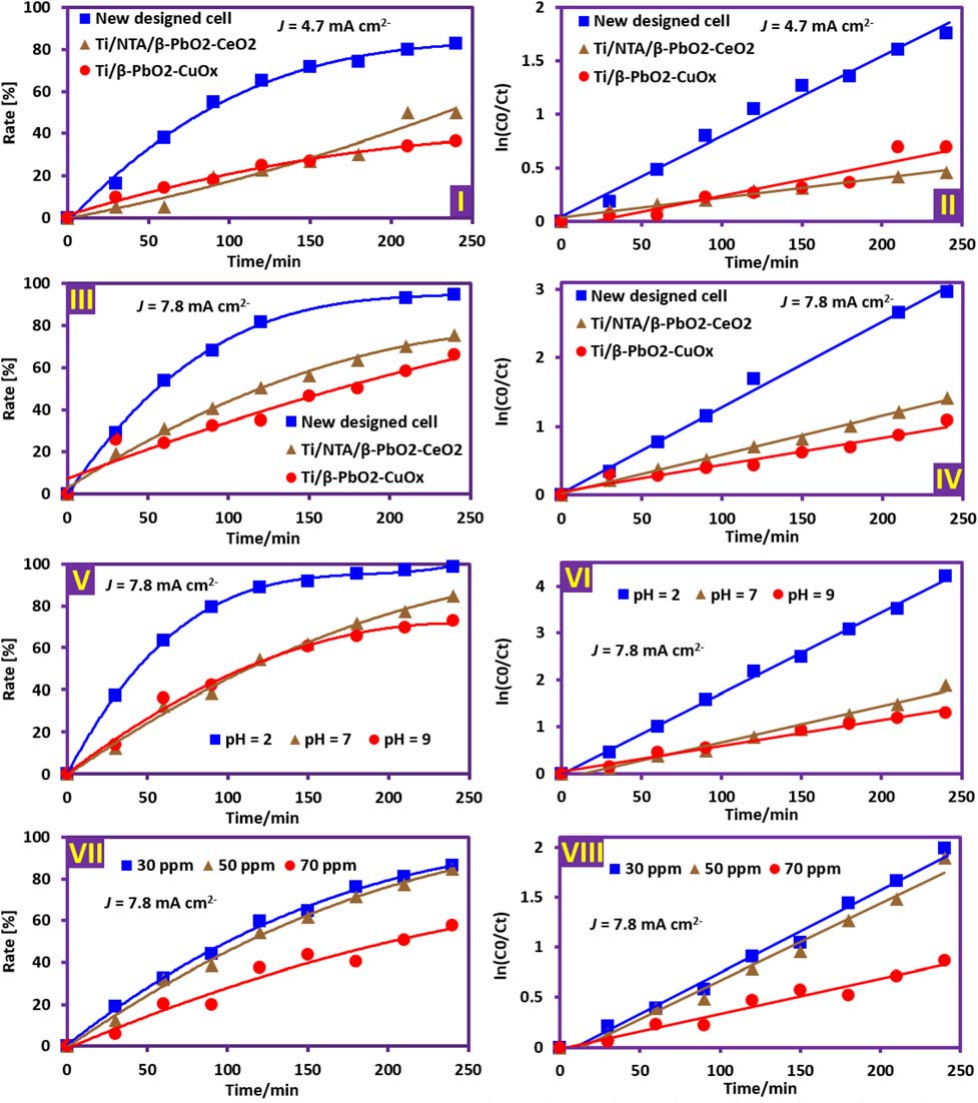
Parameters affecting the electrochemical degradation of PQ^2+^. Parts I–IV: The effect of current density and cell type. Initial PQ^2+^ concentration of 50 ppm at pH 7 in both conventional and newly designed cells. Parts V and VI: The effect of solution pH. Initial PQ^2+^ concentration of 50 ppm; applied current density 7.8 mA cm^−2^ in the newly designed cell. Parts VII and VIII: The effect of initial PQ^2+^ concentration. Solution pH 7; applied current density 7.8 mA cm^−2^ in newly designed cell.

The electrochemical degradation of PQ^2+^ was investigated using two Ti/NTA/β-PbO_2_-CeO_2_ and Ti/β-PbO_2_-CuO_*x*_ electrodes at two current densities of 4.7 and 7.8 mA cm^−2^ and at an initial concentration of 50 ppm in conventional and newly designed cells (Fig. 12 parts I–IV). The results show that in both electrodes, the degradation efficiency and degradation rate increase with increasing current density. As the current density increases, the generation of hydroxyl radicals increases, and as a result, the pollution degradation increases. The formation of more intermediates with increasing PQ^2+^ concentration and their competition with PQ^2+^ causes the degradation efficiency to decrease with increasing concentration.^18^

**Fig. 13:**
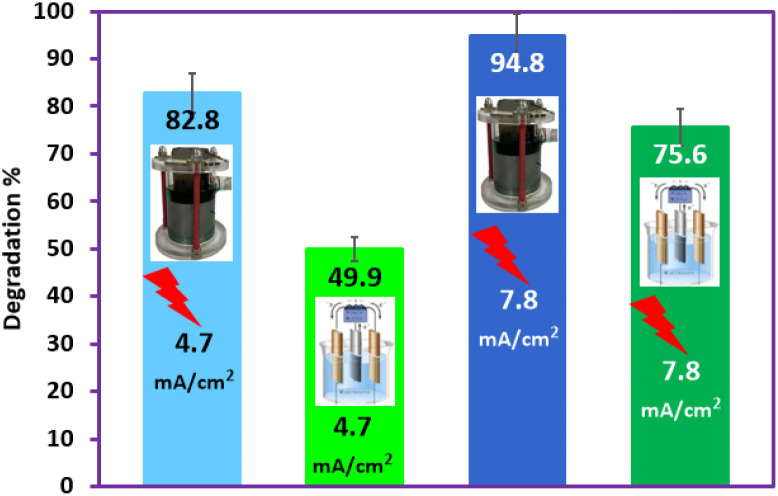
Comparison of the performance of the conventional cell (con.) with the newly designed cell (new) at two current densities of 4.7 and 7.8 mA cm^−2^ in the degradation of 50 ppm of PQ^2+^ at pH 7.

Fig. 12 parts I–IV also compares the efficiency of Ti/NTA/β-PbO_2_-CeO_2_ and Ti/β-PbO_2_-CuO_*x*_ electrodes in a conventional cell for PQ^2+^ degradation and shows that the Ti/NTA/β-PbO_2_-CeO_2_ electrode exhibits higher efficiency, especially at high current densities. Fig. 12, parts I–IV, also compares the efficiency of the new cell with a conventional cell in PQ^2+^ degradation and shows that the efficiency of the new cell is significantly higher than that of the conventional cell (Fig. 13). It is worth noting that the Ti/NTA/β-PbO_2_-CeO_2_ electrode was used in both cells.

### Influence of solution pH and PQ^2+^ initial concentration

The effect of pH value on the electrochemical degradation of PQ^2+^ was investigated at pH values of 2, 7 and 9 using the Ti/NTA/β-PbO_2_-CeO_2_ electrode at a current density of 7.8 mA cm^−2^ and an initial concentration of 50 ppm (Fig. 12 parts V and VI). The results show that the degradation efficiency is higher in an acidic environment and decreases with increasing pH. Increasing the competition between the oxygen evolution reaction and hydroxyl radical generation with increasing solution pH is the main factor in decreasing the degradation efficiency in neutral and alkaline solutions. On the other hand, hydroxyl radicals exhibit a weak acid behavior (p*K*_a_ 11.9 (ref. 42)) and therefore, in alkaline environments, they can react with hydroxyl ions (eqn (2)) and become less powerful oxidizing species.^39,43^ Although the degradation efficiency of PQ^2+^ after 240 min at pH values of 2, 7, and 9 is 98.5, 84.9, and 72.9%, respectively, pH 7 was chosen as the optimum pH due to its high compatibility with the environment.

2 HO˙ + HO^−^ → H_2_O + O˙^−^

Fig. 12, parts VII and VIII, shows the effect of PQ^2+^ initial concentration on PQ^2+^ degradation by the Ti/NTA/β-PbO_2_-CeO_2_ electrode in a new cell at a current density of 7.8 mA cm^−2^ at pH 7. As can be seen, the degradation efficiency and degradation rate decrease with increasing initial concentration of PQ^2+^.

### Influence of electrode type

In this section, the efficiency of fabricated electrodes in PQ^2+^ degradation at both current densities 4.7 and 7.8 mA cm^−2^ is compared (Fig. 12). The results show that the percentage of PQ^2+^ degradation after 240 minutes of electrolysis with the Ti/β-PbO_2_-CuO_*x*_ electrode at the current density of 4.7 and 7.8 mA cm^−2^ is 36 and 66%, respectively; however, in such conditions, the Ti/NTA/β-PbO_2_-CeO_2_ electrode has a greater efficiency, such that the percentage of PQ^2+^ degradation in the current density of 4.7 and 7.8 mA cm^−2^ becomes 49 and 75%, respectively. The better performance of the Ti/NTA/β-PbO_2_-CeO_2_ electrode in PQ^2+^ degradation can be attributed to the larger surface area and more active sites for hydroxyl radical generation than the Ti/β-PbO_2_-CuO_*x*_ electrode. In the newly designed cell, due to the large surface area of the electrodes and the surrounding anodes with a cylindrical cathode, the mass transfer is higher and the ohmic drop (*IR*) is lower. These features enable better electrochemical degradation of PQ^2+^ compared to conventional cells. Analysis of available data shows a linear relationship between time and ln(*C*_0_/*C*_*t*_), confirming that the electrochemical degradation of PQ^2+^ with the electrodes fabricated in this research follows pseudo-first-order kinetics.^18^ To obtain the necessary data, the first-order pseudo-model with the following eqn (3) has been used, where *t* is the time and *C*_0_ and *C*_*t*_ are the concentrations at the initial or a given time (ppm), respectively. *k* is the kinetic constant of the pseudo-first-order reaction, which is obtained from the slope of the plot of ln(*C*_0_/*C*_*t*_) *versus* time (*t*). The data obtained under different conditions are summarized in Table 1.

3 ln(*C*_0_/*C*_*t*_) = −*kt*

**Table 1:** Observed degradation rate constant (*k*_obs_) of PQ^2+^ under different conditions

Electrode	Current density (mA cm^−2^)	Initial concentration (ppm)	pH value	*R* ^2^	*K*_obs_ (min^−1^)
Ti/NTA/β-PbO_2_-CeO_2_	4.7	50	7	0.9136	0.0030
Ti/NTA/β-PbO_2_-CeO_2_	7.8	50	7	0.9968	0.0057
Ti/β-PbO_2_-CuO_*x*_	4.7	50	7	0.9821	0.0018
Ti/β-PbO_2_-CuO_*x*_	7.8	50	7	0.9531	0.0040
Ti/NTA/β-PbO_2_-CeO_2_	7.8	50	2	0.9974	0.0172
Ti/NTA/β-PbO_2_-CeO_2_	7.8	50	7	0.9836	0.0077
Ti/NTA/β-PbO_2_-CeO_2_	7.8	50	9	0.9884	0.0055
Ti/NTA/β-PbO_2_-CeO_2_	7.8	30	7	0.9898	0.0082
Ti/NTA/β-PbO_2_-CeO_2_	7.8	70	7	0.9616	0.0035
Ti/NTA/β-PbO_2_-CeO_2_ in new cell	4.7	50	7	0.9858	0.0075
Ti/NTA/β-PbO_2_-CeO_2_ in new cell	7.8	50	7	0.9953	0.0125

### Energy consumption

In this section, the effects of electrode type, applied current density, and cell type on electrical energy consumption (EEC) in the electrochemical degradation of PQ^2+^ is investigated. Here, EEC is described by the EE/O (electrical efficiency per log order). EE/O is defined as the electricity consumed by reducing the concentration of the pollutant by an order of magnitude. Eqn (4) is used to calculate electrical efficiency per log order (EE/O):^46^

4 EE/O (kWh m^−3^) = 0.001*EIt*/*V*_s_ log(*C*_0_/*C*_*t*_)

Here, *E* is the cell voltage (V), *I* is the applied current (A), *t* is the electrolysis time (hour), *C*_0_ is the initial concentration of PQ^2+^, *C*_F_ is the final concentration of PQ^2+^ and *V*_s_ is the cell volume (m^3^). The data of EE/O are listed in Table 2. These results indicate that the electrode type does not have a significant effect on EE/O; however, in both electrodes, EE/O increases with increasing current density. The occurrence of oxygen evolution reaction at a higher current density seems to be one of the important factors in increasing EE/O. Furthermore, at higher current densities, the process of converting hydroxyl radicals into hydrogen peroxide and hydroperoxyl radical increases (eqn (5) and (6)).^44^ It should be noted that since the oxidizing power of H_2_O_2_ and 
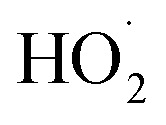
 is much lower than that of HO˙,^43^ these compounds reduce the mineralization process and, in other words, increase EE/O. Unlike the electrode type, the cell type has a significant impact on EE/O, thus, using the cell designed in this study reduces EE/O by approximately 2.5 times.

5 2HO˙ → H_2_O_2_

6
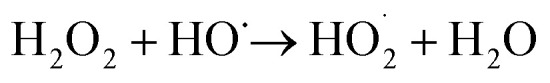


**Table 2:** EE/O and OPC values for the degradation of PQ^2+^ under different conditions^a^

Electrode	Current density (mA cm^−2^)	Cell volume (m^3^)	Cell voltage (V)	log(*C*_0_/*C*_F_)	EE/O (kWh m^−3^)	OPC^b^ US$ per m^3^
Ti/NTA/β-PbO_2_-CeO_2_	7.8	0.08	10.4	0.61	0.42	3.48
Ti/NTA/β-PbO_2_-CeO_2_	4.7	0.08	9.3	0.30	0.46	3.49
Ti/β-PbO_2_-CuO_*x*_	7.8	0.08	7.5	0.47	0.40	3.48
Ti/β-PbO_2_-CuO_*x*_	4.7	0.08	6.4	0.20	0.48	3.49
Ti/NTA/β-PbO_2_-CeO_2_ in new cell	7.8	0.17	9.0	1.28	0.18	3.46
Ti/NTA/β-PbO_2_-CeO_2_ in new cell	4.7	0.17	8.2	0.76	0.16	3.46

a PQ^2+^ initial concentration: 50 ppm, initial pH = 7.0 and electrolysis time of 240 min.

b The cost of electrodes is not included.

The operating cost (OPC) of an electrochemical treatment process depends mainly on the cost of energy consumed under optimal conditions and the cost of electrodes and chemicals. The equation for the calculation of operating cost is shown below:^45^

7 OPC = *α* × ENC + *β* × CHC + *γ* × ELC

where ENC, CHC and, ELC are energy consumption per cubic meter of wastewater (kWh m^3^), cost of chemicals (kg m^−3^) and consumed electrode for treatment of a cubic meter wastewater (kg m^−3^), respectively. *α* is the price of electricity, *β* is the price of chemicals, and *γ* is the price of electrodes.

Electricity price (*α*): 0.1 US$ per kWh and therefore: ENC for Ti/NTA/β-PbO_2_-CeO_2_ in the new cell is: 0.16 × 0.1 = 0.016 US$ per m^3^. The cost of phosphate buffer (*β*) is 3.44 US$ per m^3^ Since in electrocatalytic degradation processes, electrodes can be used for a long time and, on the other hand, only a thin layer of catalysts and chemicals is deposited on them (the prepared chemicals and solutions can be used several times), the cost of the electrodes is negligible and has not been included in these calculations.^46^

Therefore:Operating cost (US$ per m^3^) = 0.016 + 3.44 = 3.46 US$ per m^3^

Other data are shown in Table 2.

### Electrochemical degradation pathway of PQ^2+^

**Scheme 2:**
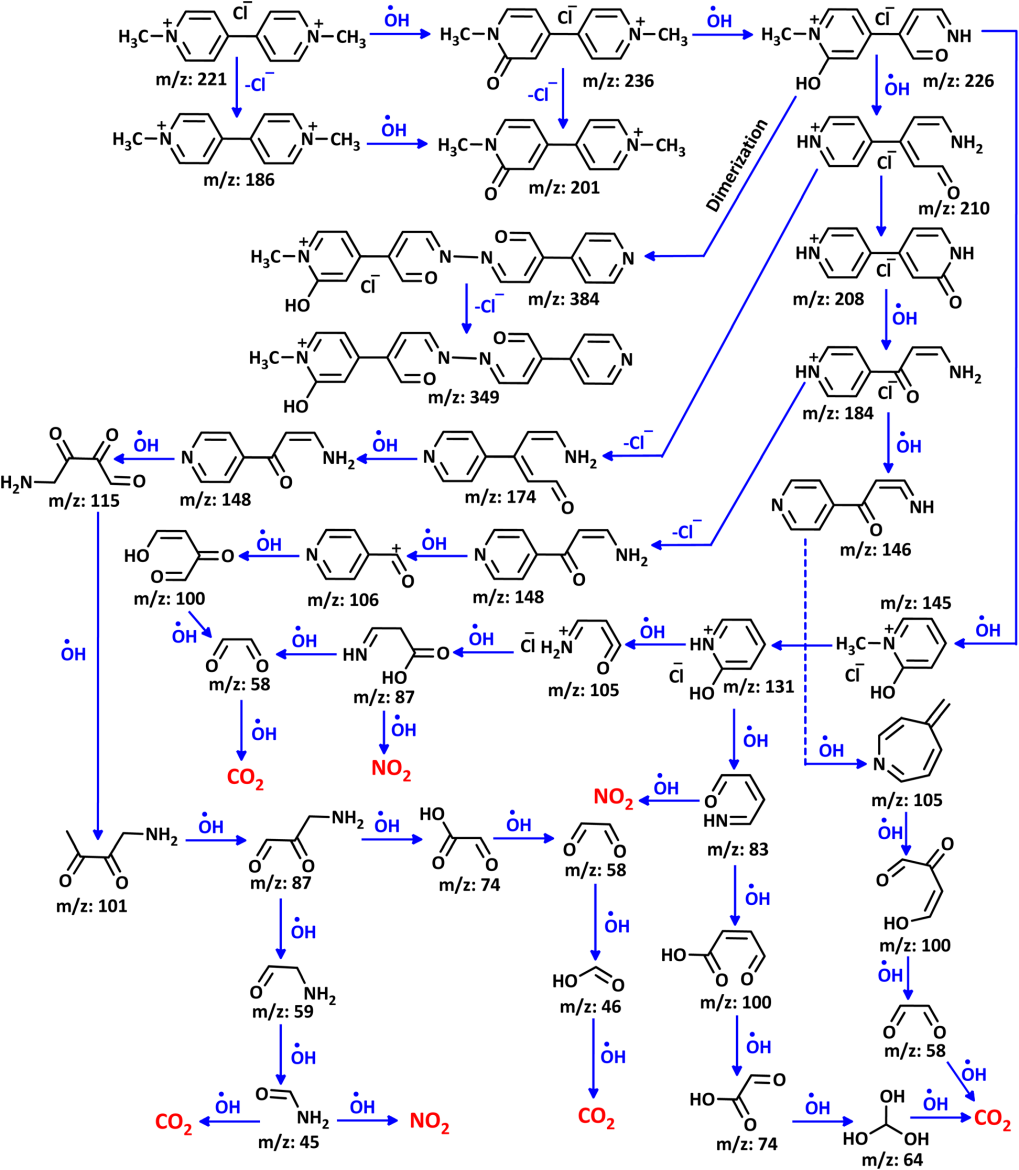
Proposed mechanism for the electrochemical degradation of PQ^2+^. PQ^2+^ initial concentration: 50 ppm. Initial pH: 7.0. Applied current density: 7.8 mA cm^−2^, in new cell at room temperature.

The proposed mechanism for the electrochemical degradation of PQ^2+^ by the Ti/NTA/β-PbO_2_-CeO_2_ electrode is shown in Scheme 2. All fragments reported in this Figure are based on data obtained from LC-MS spectra of PQ^2+^ solution during electrolysis (SI). It seems that the first step in the electrochemical degradation of PQ^2+^ is the oxidation/hydroxylation/ring opening and demethylation of PQ^2+^ by OH˙, resulting in the formation of C_12_H_13_ClN_2_O (*m*/*z* = 236) and C_10_H_11_ClN_2_O_2_ (*m*/*z* = 226), respectively.^14^

Dimerization of the fragment C_10_H_11_ClN_2_O_2_ (*m*/*z* = 226), in the ionization chamber of the mass spectrometer or during electrolysis leads to C_19_H_17_ClN_4_O_3_ (*m*/*z* = 384) in the early stages. In parallel, demethylation/oxidation and degradation of this compound produced fragments C_8_H_9_ClN_2_O (*m*/*z* = 184) and C_8_H_6_N_2_O (*m*/*z* = 146). Oxidation/degradation and rearrangement of C_8_H_6_N_2_O lead to fragments C_7_H_7_N (*m*/*z* = 105), C_4_H_4_O_3_ (*m*/*z* = 100), and C_2_H_2_O_2_ (*m*/*z* = 58), respectively. Further oxidation of these fragments eventually leads to CO_2_ and NO_2_. As shown in Scheme 2, the same steps and processes ultimately lead to the formation of CO_2_ and NO_2_ in other pathways.

**Scheme 3:**
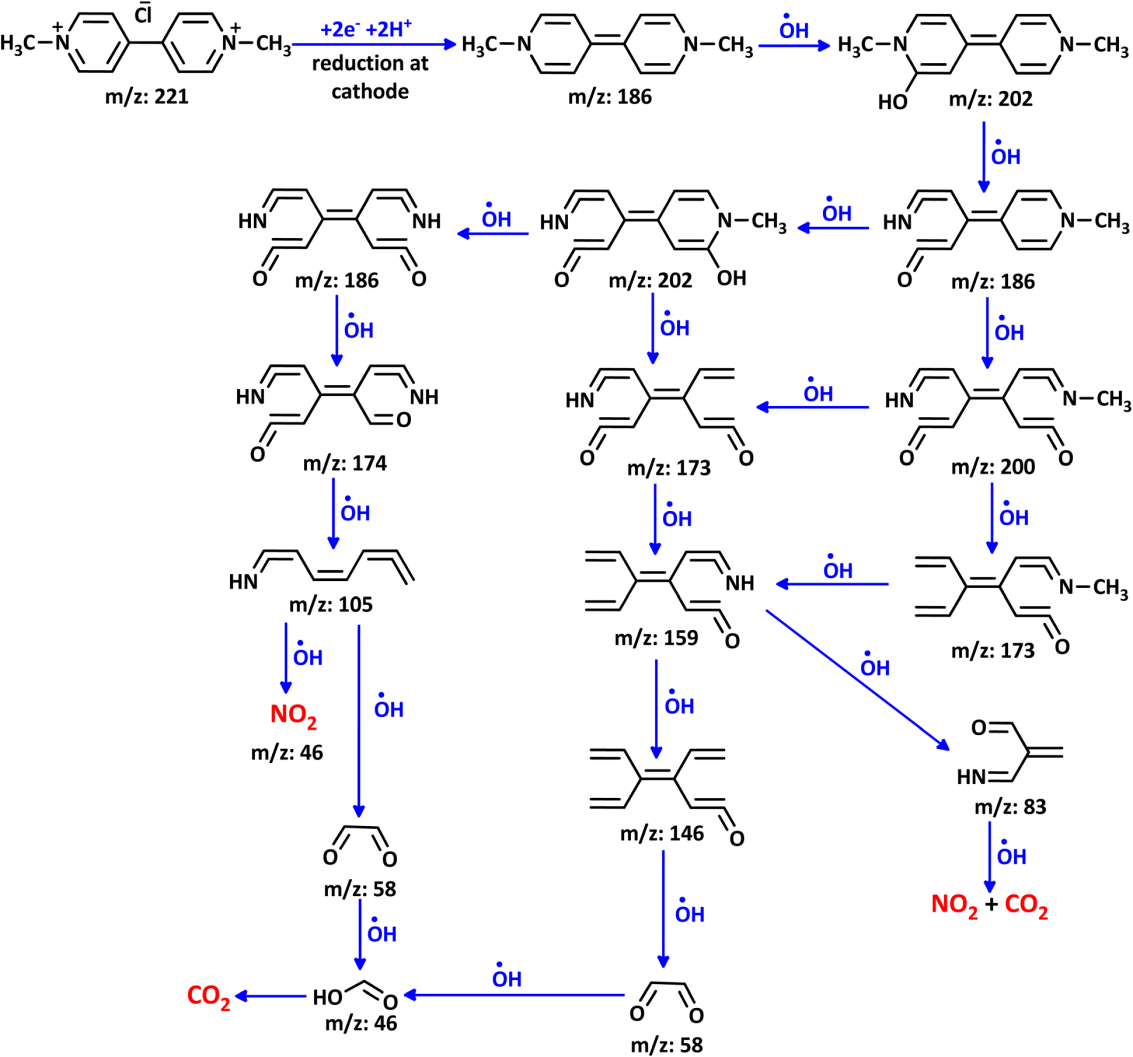
Proposed mechanism for the degradation of PQ^2+^ based on reduction.

In addition to the mechanism proposed in Scheme 2, voltammetric results (Fig. 10) indicate that PQ^2+^ can be reduced to PQ at the cathode surface. The reduced molecule (PQ), due to its conjugated double bonds, can react with hydroxyl radicals^47^ to form a molecule with a mass of 202 (Scheme 3). Subsequent oxidation and addition by hydroxyl radicals transforms this molecule into the intermediates shown in Scheme 3, which are eventually mineralized. It should be noted that all fragments reported in Scheme 3 are based on data obtained from the LC-MS spectra of the PQ^2+^ solution during electrolysis (SI).

## Conclusion

In this research, two types of electrodes, Ti/NTA/β-PbO_2_-CeO_2_ and Ti/β-PbO_2_-CuO_*x*_, were fabricated and used for the successful degradation of PQ^2+^. The results showed that the Ti/NTA/β-PbO_2_-CeO_2_ electrode increases the production of hydroxyl radicals and is more efficient in PQ^2+^ degradation than the Ti/β-PbO_2_-CuO_*x*_ electrode. The most important factor seems to be the doping of β-PbO_2_ with CeO_2_. CeO_2_ doping makes the β-PbO_2_ grains finer, which leads to improved grain arrangement and an increase in the number of active sites for the hydroxyl radical production. Using this electrode, we were able to remove up to 75% of PQ^2+^ at pH 7, current density of 7.8 mA cm^−2^ and an initial concentration of 50 ppm. The important innovation of this study is the design of a new electrochemical cell, the use of which, due to its special design, increases the degradation efficiency by 95% and reduces energy consumption by 40%. Another interesting point of this research is the provision of a complete and detailed mechanism for the degradation of PQ^2+^ using data obtained from LC-MS experiments and the identification of different mineralization pathways for this compound. In this study, the electrochemical behavior of PQ^2+^ was also investigated using cyclic voltammetry in different water/acetonitrile mixtures. Our data show that the reduced form of PQ^2+^ (PQ) has strong adsorption properties in neutral and alkaline solutions, which should be considered in environmental assessments. These results will lead to deeper insights and understanding of the redox properties and adsorption activity of PQ^2+^. Finally, given the ability of the current strategy to the degradation of the robust chemical structure of PQ^2+^, it seems that this method is capable of degrading a wide range of organic pollutants. Also, the proposed method can be easily scaled up by increasing the dimensions of the designed cell.

## Ethical statement

This article does not contain any studies with animals performed by any of the authors.

## Author contributions

Davood Nematollahi: supervision, project administration, resources, writing-review and editing. Mahsa Roshani: investigation, formal analysis, and writing-original draft. Mohammad Mehdi Hashemi-Mashouf: investigation. Niloofar Mohamadighader: investigation.

## Conflicts of interest

The authors declare no conflict of interest.

## Supplementary Material

RA-015-D5RA06796K-s001

## Data Availability

All data generated or analyzed during this study are included in this published article and its supplementary information (SI) files. Supplementary information is available. See DOI: https://doi.org/10.1039/d5ra06796k.

## References

[cit1] Zou T., He P., Cao J., Li Z. (2015). Determination of paraquat in vegetables using HPLC-MS-MS. J. Chromatogr. Sci..

[cit2] Sieliechi J., Thue P. (2015). Removal of paraquat from drinking water by activated carbon prepared from waste wood, Desalin. Water Treat..

[cit3] Tsai W. T., Lai C. W., Hsien K. J. (2004). Adsorption kinetics of herbicide paraquat from aqueous solution onto activated bleaching earth. Chemosphere.

[cit4] Keawkumay C., Rongchapo W., Sosa N., Suthirakun S., Koleva I. Z., Aleksandrov H. A., Vayssilov G. N., Wittayakun J. (2019). Paraquat adsorption on NaY zeolite at various Si/Al ratios: A combined experimental and computational study. Mater. Chem. Phys..

[cit5] Hsu S. T., Pan T. C. (2007). Adsorption of paraquat using methacrylic acid-modified rice husk. Bioresour. Technol..

[cit6] Seki Y., Yurdakoç K. (2005). Paraquat adsorption onto clays and organoclays from aqueous solution. J. Colloid Interface Sci..

[cit7] Han Y. S., Lee S. Y., Yang J. H., Soo Hwang H., Park I. (2010). Paraquat release control using intercalated montmorillonite compounds. J. Phys. Chem. Solids.

[cit8] Kang M. (2002). Preparation of TiO_2_ photocatalyst film and its catalytic performance for 1,1′-dimethyl-4,4′-bipyidium dichloride decomposition. Appl. Catal., B.

[cit9] Kearney P. C., Ruth J. M., Zeng Q., Mazzocchi P. (1985). UV ozonation of paraquat. J. Agric. Food Chem..

[cit10] Roshani M., Nematollahi D., Ansari A., Adib K., Masoudi-Khoram M. (2024). Boosted electrocatalytic oxidation of organophosphorus pesticides by a novel high-efficiency CeO_2_-Doped PbO_2_ anode: An electrochemical study, parameter optimization and degradation mechanisms. Chemosphere.

[cit11] Dhaouadi A., Adhoum N. (2009). Degradation of paraquat herbicide by electrochemical advanced oxidation methods. J. Electroanal. Chem..

[cit12] Cartaxo M. A. M., Borges C. M., Pereira M. I. S., Mendonça M. H. (2015). Electrochemical oxidation of paraquat in neutral medium. Electrochim. Acta.

[cit13] Tadayozzi Y. S., Santos F. A. d., Vicente E. F., Forti J. C. (2021). Application of oxidative process to degrade paraquat present in the commercial herbicide. J. Environ. Sci. Health, Part B.

[cit14] Khlifi H., Guesmi A., Rabaaoui N., Cherif M., Mhadhbi N., Abd El-Fattah W., Hamadi N. B., Naïli H. (2025). From molecular architecture to environmental action: a new palladium-based perovskite catalyst as a cathodic modifier for electro-Fenton degradation. RSC Adv..

[cit15] Teutli-Sequeira E. A., Vasquez-Medrano R., Prato-Garcia D., Ibanez J. G. (2024). The electrooxidation of synthetic bipyridyl herbicide wastewaters with boron-doped diamond electrodes: A technical and economic study to boost. Their application for pollution prevention in the agricultural sector. Processes.

[cit16] Bautista-García B. Y., Castillo-Suárez L. A., Teutli-Sequeira E. A., Castañeda-Juárez M., Linares-Hernández I., Martínez-Miranda V. (2025). Degradation of the commercial paraquat herbicide by UVA-LED photo-electrooxidation utilizing a BDD-Fe system: Multiple response optimization. Water, Air, Soil Pollut..

[cit17] Rabaaoui N., Hamadi N. B., Cherif M., Guesmi A., Abd El-Fattah W., Naïli H. (2025). Anodic oxidation of paraquat herbicide on BDD electrode: comparative evaluation of variable effects and degradation mechanisms. RSC Adv..

[cit18] Roshani M., Nematollahi D., Hashemi-Mashouf M. M., Mohamadighader N., Ansari A. (2024). Highly efficient electrocatalytic degradation of methylparaben using BiO_x_-doped Ti/β-PbO_2_ anode: Comprehensive electrochemical study and degradation mechanism. Electrochim. Acta.

[cit19] Hao X., Wuqi G., Jia W., Jiangtao F., Honghui Y., Wei Y. (2016). Preparation and characterization of titanium-based PbO_2_ electrodes modified by ethylene glycol. RSC Adv..

[cit20] Marien C. B. D., Cottineau T., Robert D., Drogui P. (2016). TiO_2_ Nanotube arrays: Influence of tube length on the photocatalytic degradation of Paraquat. Appl. Catal., B.

[cit21] Gong D., Grimes C. A., Varghese O. K., Hu W., Singh R., Chen Z., Dickey E. C. (2001). Titanium oxide nanotube arrays prepared by anodic oxidation. J. Mater. Res..

[cit22] Cai Q., Paulose M., Varghese O. K., Grimes C. A. (2005). The effect of electrolyte composition on the fabrication of self-organized titanium oxide nanotube arrays by anodic oxidation. J. Mater. Res..

[cit23] Morales J., Petkova G., Cruz M., Caballero A. (2004). Nanostructured lead dioxide thin electrode. Electrochem. Solid-State Lett..

[cit24] Lyu J., Han H., Wu Q., Ma H., Ma C., Dong X., Fu Y. (2019). Enhancement of the electrocatalytic oxidation of dyeing wastewater (reactive brilliant blue KN-R) over the Ce-modified Ti-PbO_2_ electrode with surface hydrophobicity. J. Solid State Chem..

[cit25] Ye K., Li Y., Yang H., Li M., Huang Y., Zhang S., Ji H. (2019). An ultrathin carbon layer activated CeO_2_ heterojunction nanorods for photocatalytic degradation of organic pollutants. Appl. Catal., B.

[cit26] Huang Y., Guo Z., Liu H., Zhang S., Wang P., Lu J., Tong Y. (2019). Heterojunction architecture of N-doped WO_3_ nanobundles with Ce_2_S_3_ nanodots hybridized on a carbon textile enables a highly efficient flexible photocatalyst. Adv. Funct. Mater..

[cit27] Acevedo-Peña P., Lartundo-Rojas L., González I. (2013). Effect of water and fluoride content on morphology and barrier layer properties of TiO_2_ nanotubes grown in ethylene glycol-based electrolytes. J. Solid State Chem..

[cit28] Wang Q., Tu S., Wang W., Chen W., Duan X., Chang L. (2021). Optimized Indium modified Ti/PbO_2_ anode for electrochemical degradation of antibiotic cefalexin in aqueous solutions. Colloids Surf., A.

[cit29] Biesinger M. C., Lau L. W. M., Gerson A. R., Smart R. S. C. (2010). Resolving surface chemical states in XPS analysis of first row transition metals, oxides and hydroxides: Sc, Ti, V, Cu and Zn. Appl. Surf. Sci..

[cit30] Chawla S., Sankarraman N., Payer J. (1992). Diagnostic spectra for XPS analysis of Cu–O–S–H compounds. J. Electron Spectrosc. Relat. Phenom..

[cit31] Wang Z., Xu M., Wang F., Liang X., Wei Y., Hu Y., Zhu C. G., Fang W. (2017). Preparation and characterization of a novel Ce doped PbO_2_ electrode based on NiO modified Ti/TiO_2_NTs substrate for the electrocatalytic degradation of phenol wastewater. Electrochim. Acta.

[cit32] Sopha H., Spotz Z., Sepúlveda M., Alijani M., Motola M., Hromadko L., Macak J. M. (2023). Intrinsic properties of anodic TiO_2_ nanotube layers: In-situ XRD annealing of TiO_2_ nanotube layers. Ceram. Int..

[cit33] Xu M., Mao Y., Song W., OuYang X., Hu Y., Wei Y., Zhu C., Fang W., Shao B., Lu R., Wang F. (2018). Preparation and characterization of Fe-Ce co-doped Ti/TiO_2_ NTs/PbO_2_ nanocomposite electrodes for efficient electrocatalytic degradation of organic pollutants. J. Electroanal. Chem..

[cit34] Dolatabadi M., Ehrampoush M. H., Pournamdari M., Ebrahimi A. A., Fallahzadeh H., Ahmadzadeh S. (2023). Enhanced electrocatalytic elimination of fenitrothion, trifluralin, and chlorothalonil from groundwater and industrial wastewater using modified Cu-PbO_2_ electrode. J. Mol. Liq..

[cit35] Shmychkova O., Luk’yanenko T., Velichenko A., Meda L., Amadelli R. (2013). Bi-doped PbO_2_ anodes: Electrodeposition and physico-chemical properties. Electrochim. Acta.

[cit36] Hao X., Dan S., Qian Z., Honghui Y., Yan W. (2014). Preparation and characterization of PbO_2_ electrodes from electro-deposition solutions with different copper concentration. RSC Adv..

[cit37] Yuzhu S., Zhen C., Lai G., Qiang Y., Wei Z., Dan W., Tao Z. (2019). Fabrication and electrocatalytic performance of a two dimensional β-PbO_2_ macroporous array for methyl orange degradation. Int. J. Electrochem. Sci..

[cit38] Reghunath S., Pinheiro D., Kr S. D. (2021). A review of hierarchical nanostructures of TiO_2_: Advances and applications. Appl. Surf. Sci. Adv..

[cit39] Ansari A., Nematollahi D. (2018). A comprehensive study on the electrocatalytic degradation, electrochemical behavior and degradation mechanism of malachite green using electrodeposited nanostructured beta-PbO_2_ electrodes. Water Res..

[cit40] Wang W., Duan X., Sui X., Wang Q., Xu F., Chang L. (2020). Surface characterization and electrochemical properties of PbO_2_/SnO_2_ composite anodes for electrocatalytic oxidation of *m*-nitrophenol. Electrochim. Acta.

[cit41] Bard A. J. and Faulkner L. R., Electrochemical Methods-Fundamentals and Applications, John Wiley & Sons, 2nd edn, 2001, p. 590

[cit42] Kisała J., Goclon J., Pogocki D. (2021). Reductive dehalogenation–challenges of perfluorinated organics. Journal of Photocatalysis.

[cit43] Moreira F. C., Boaventura R. A. R., Brillas E., Vilar V. J. P. (2017). Electrochemical advanced oxidation processes: A review on their application to synthetic and real wastewaters. Appl. Catal., B.

[cit44] Bian X., Xia Y., Zhan T., Wang L., Zhou W., Dai Q., Chen J. (2019). Electrochemical removal of amoxicillin using a Cu doped PbO_2_ electrode: Electrode characterization, operational parameters optimization and degradation mechanism. Chemosphere.

[cit45] Hashemi-Mashouf M. M., Nematollahi D., Alaei M. (2025). Comparative degradation of amido black 10B and Bismarck brown by electro-Fenton process. Comprehensive electrochemical study and degradation pathway of amido black 10B. Results Eng..

[cit46] Regalado-Méndez A., Zavaleta-Avendaño J., Alanis-Ramírez C., Amado-Piña D., Ramírez Serrano A., Peralta-Reyes E. (2024). Electrochemical mineralization of chloroquine in a filter-press-type flow reactor in batch recirculation mode equipped with two boron-doped diamond electrodes: parametric optimization, total operating cost, phytotoxicity test, and life cycle assessment. Catalysts.

[cit47] Malmir M., Nematollahi D., Sadatnabi A., Shanehsaz S. (2025). Electrochemically induced Meerwein arylation as a green strategy for the synthesis of arylbenzoquinone derivatives under batch and flow conditions. Sci. Rep..

