# Supplementary material for: Applications of Ti/TiO2 nanotube arrays/CeO2@PbO2 and Ti/β-PbO2-CuOx electrodes for efficient electrocatalytic degradation of paraquat: cell design and comprehensive mechanistic studies
Source: RSC Adv. 2025 Oct 17;15(46):39148–60. doi: 10.1039/d5ra06796k (PMC12531992; doi:10.1039/d5ra06796k)
Supplement: RA-015-D5RA06796K-s001 [file RA-015-D5RA06796K-s001.pdf]

Supplementary Information (SI) for RSC Advances.
This journal is © The Royal Society of Chemistry 2025

Supplementary data

Applications of Ti/TiO_2 nanotube arrays/CeO_2@PbO_2 and Ti/β-PbO_2-CuO_x electrode for efficient electrocatalytic degradation of paraquat. Cell design and comprehensive mechanistic studies

Davood Nematollahi, [a,b]* Mahsa Roshani,[a] Mohammad mehdi Hashemi-Mashouf,[a] Niloofar Mohamadighader[a]

[a]Faculty of Chemistry and Petroleum Sciences, Bu-Ali Sina University, Hamedan 6517838683, Iran.
[b]Planet Chemistry Research Center, Bu-Ali Sina University, Hamedan, Iran.

*Corresponding author. Tel.: + 0098 813 8271541; fax: +0098 813 8272404.
E-mail addresses: nemat@basu.ac.ir, dnematollahi@yahoo.com (D. Nematollahi).
Fax: +0098 813 8257407, Tel: +0098 813 8282807.

LC-MS spectra of PQ^{++} degradation by *Ti/NTA/β-PbO₂-CeO₂* anode.

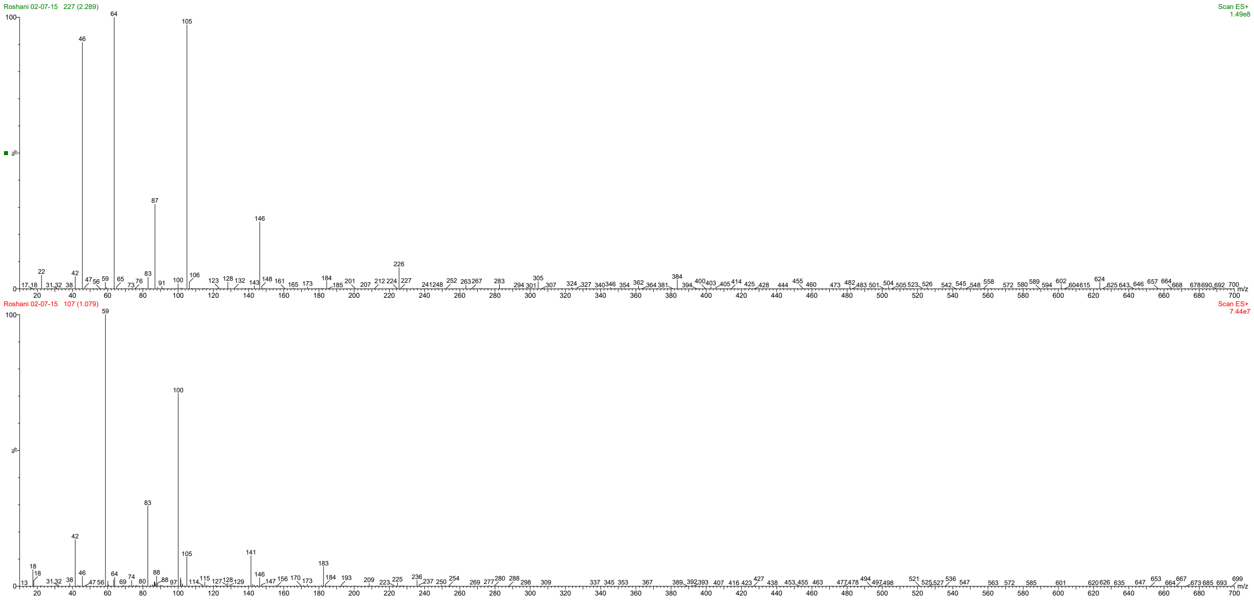

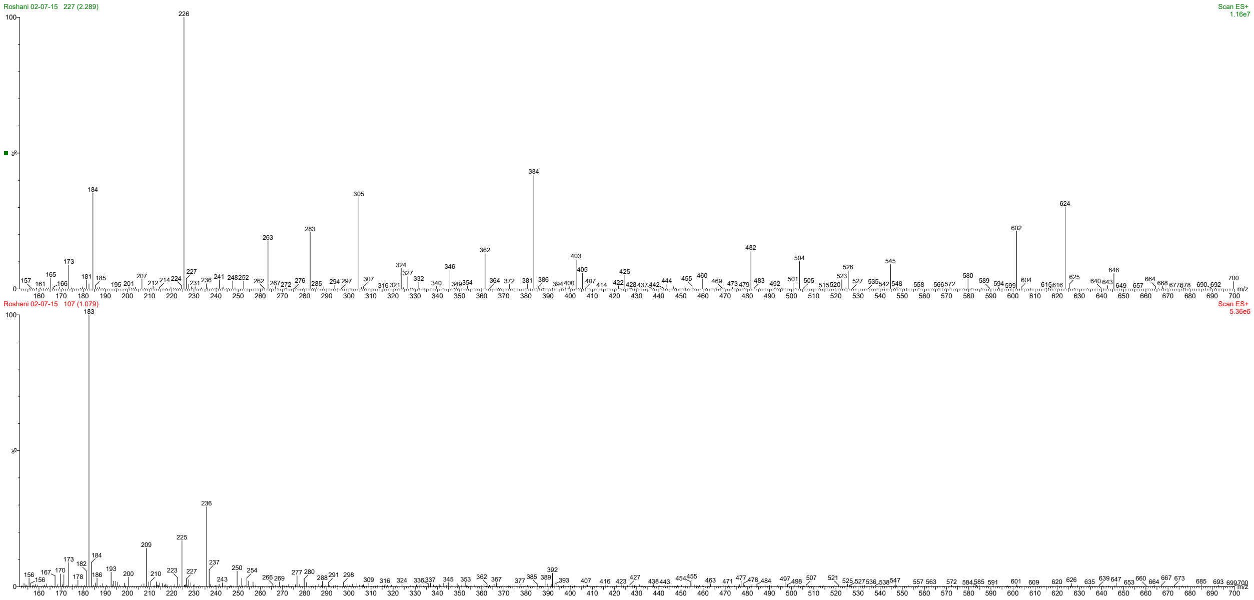

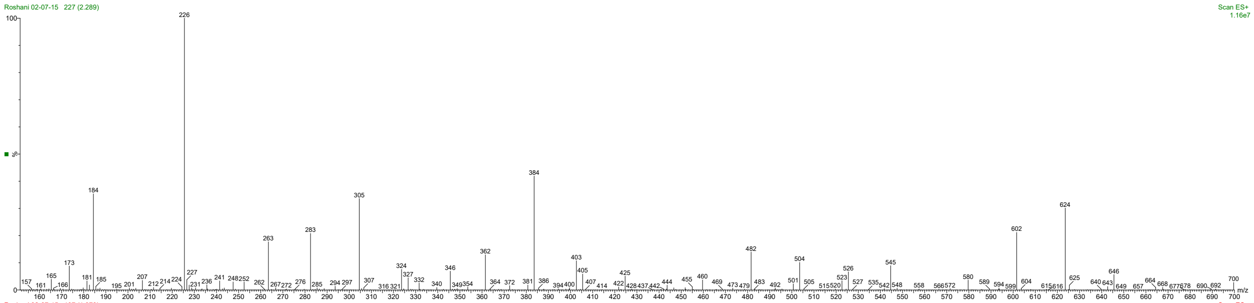
Roshani 02-07-15 227 (2.289)
Scan ES+
1.16e7

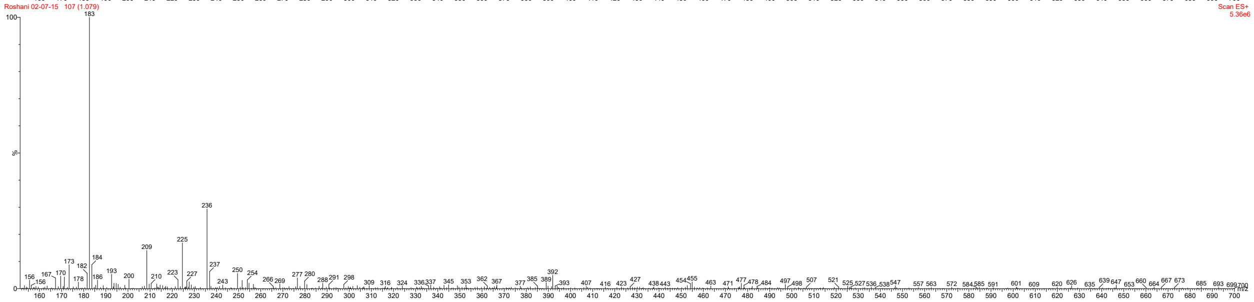
Roshani 02-07-15 107 (1.079)
Scan ES+
5.36e6

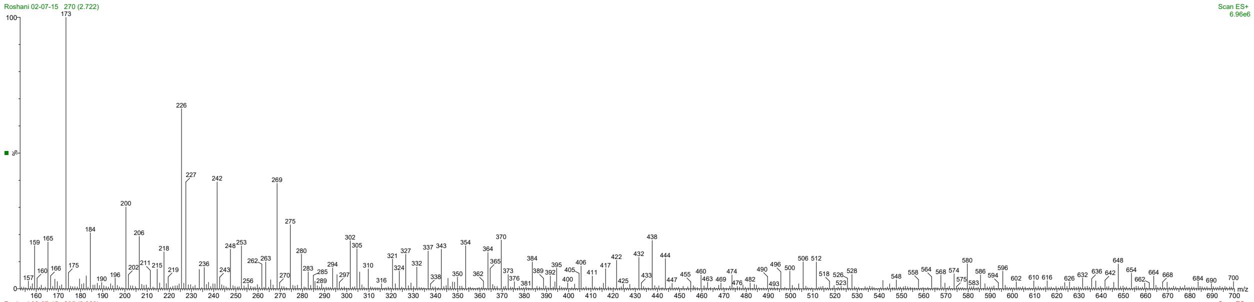
Roshani 02-07-15 270 (2.722)
Scan ES+
6.96e6

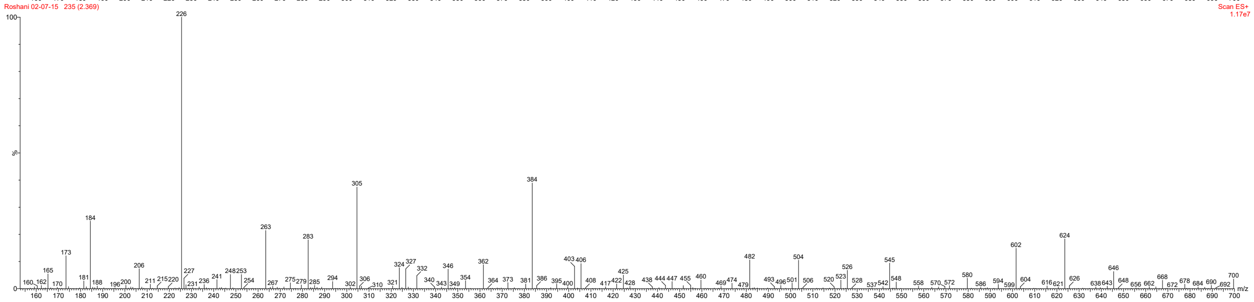
Roshani 02-07-15 235 (2.369)
Scan ES+
1.17e7
